# Macronutrient Quality and All-Cause Mortality in the SUN Cohort

**DOI:** 10.3390/nu13030972

**Published:** 2021-03-17

**Authors:** Susana Santiago, Itziar Zazpe, Cesar I. Fernandez-Lazaro, Víctor de la O, Maira Bes-Rastrollo, Miguel Ángel Martínez-González

**Affiliations:** 1 Department of Nutrition and Food Sciences and Physiology, Campus Universitario, School of Pharmacy and Nutrition, University of Navarra, 31008 Pamplona, Spain; ssantiago@unav.es (S.S.); izazpe@unav.es (I.Z.); 2 Department of Preventive Medicine and Public Health, Campus Universitario, School of Medicine, University of Navarra, 31008 Pamplona, Spain; cflazaro@unav.es (C.I.F.-L.); vdelao@alumni.unav.es (V.d.l.O.); mbes@unav.es (M.B.-R.)

**Keywords:** macronutrient quality, carbohydrates, fat, proteins, SUN cohort, all-cause mortality

## Abstract

No previous study has assessed the relationship between overall macronutrient quality and all-cause mortality. We aimed to prospectively examine the association between a multidimensional macronutrient quality index (MQI) and all-cause mortality in the SUN (Seguimiento Universidad de Navarra) (University of Navarra Follow-Up) study, a Mediterranean cohort of middle-aged adults. Dietary intake information was obtained from a validated 136-item semi-quantitative food-frequency questionnaire. We calculated the MQI (categorized in quartiles) based on three quality indexes: the carbohydrate quality index (CQI), the fat quality index (FQI), and the healthy plate protein source quality index (HPPQI). Among 19,083 participants (mean age 38.4, 59.9% female), 440 deaths from all causes were observed during a median follow-up of 12.2 years (IQR, 8.3–14.9). No significant association was found between the MQI and mortality risk with multivariable-adjusted hazard ratio (HR) for the highest vs. the lowest quartile of 0.79 (95% CI, 0.59–1.06; P_trend_ = 0.199). The CQI was the only component of the MQI associated with mortality showing a significant inverse relationship, with HR between extreme quartiles of 0.64 (95% CI, 0.45–0.90; P_trend_ = 0.021). In this Mediterranean cohort, a new and multidimensional MQI defined a priori was not associated with all-cause mortality. Among its three sub-indexes, only the CQI showed a significant inverse relationship with the risk of all-cause mortality.

## 1. Introduction

Healthy eating results essential to reduce the risk of developing non-communicable diseases and increases the number of years lived with good health [1]. However, the potential impact of diet quality on chronic diseases and mortality is frequently underestimated. Findings of the Global Burden of Disease Study suggested that dietary risks were responsible for approximately 22% of all adult deaths (11 million deaths), which calls for an action to improve overall dietary quality intake [2].

Although diet quality is a complex concept that is difficult to measure, general recommendations for a healthy eating include fostering bioactive-rich foods (vegetables, fruits, seeds, whole grains, nuts, plant oils, fish, and yogurt), selecting non-processed or minimally-processed foods and avoiding ultra-processed products, which are important sources of sugars, refined starch, and industrial additives such as trans fatty acids and sodium [3]. In this context, multiple dietary indices have gained attention to measure diet quality. These indices are used in nutritional epidemiology to assess compliance with national nutrition guidelines, adherence to predefined high-quality or healthy dietary patterns, dietary changes and risk of developing chronic diseases [4]. Diet quality indices usually include items related to nutrients, foods/food groups or, most frequently, a combination of both. Diet quality indices differ in the number of foods and nutrients included, the cut-off values, and the scoring algorithms used to build them [5].

In general, an adequate balance of macronutrients and micronutrients is a common dimension of diet quality, which has been recently included in several emerging indices. Specifically, the proportionality of the energy-yielding macronutrients (proteins, carbohydrates, and fats) and/or fatty acids (monounsaturated, polyunsaturated, saturated, and trans fatty acids) (MUFA, PUFA, SFA and TFA) has been mainly addressed by preferred intake ranges and intake ratios for fatty acids [6]. Additionally, nutritional dietary guidelines across countries have proposed ranges of energy macronutrient distribution to improve overall health and prevent chronic diseases [7]. However, optimal macronutrient composition for supporting longevity remains uncertain [8,9]. In fact, some authors [10] have investigated global associations between macronutrient supply and age-specific mortality, concluding that the macronutrient composition of energy supply that minimizes mortality varies with age.

Regarding energy from carbohydrates, both high and low percentages, have been associated with increased mortality, observing the lowest risk of mortality between 50–55% of calories from carbohydrate intake [11]. In the PURE Study, the authors found that energy intake from carbohydrates higher than 60% was associated with total mortality risk, whereas no association was found for total fat and types of fat [12]. Growing evidence on dietary fats, particularly saturated fats, in relation to health and mortality is also conflicting [13,14]. 

Evidence from prospective cohort studies suggests that animal protein intake is associated with increased all-cause mortality, as opposed to plant protein, whereas evidence of the effect of total protein intake is more inconsistent [15,16,17]. 

Beyond intake or percentage of total energy intake, the potential association of macronutrients with mortality ultimately relies on overall dietary quality and specific food sources [9,18]. However, quality of macronutrients, primarily carbohydrate quality intake, has been investigated in relation to mortality [19], chronic diseases [20,21], and inflammatory markers [22]. To the best of our knowledge, to date, there is no study that has specifically investigated the association between global macronutrient quality intake and all-cause mortality. Hence, we aimed to assess the association of global macronutrient quality, through a multidimensional macronutrient quality index (MQI) with all-cause mortality in the “Seguimiento Universidad de Navarra (SUN)” (University of Navarra Follow-Up) Project.

## 2. Materials and Methods

### 2.1. Study Design

The SUN Project is a Spanish dynamic, multipurpose, prospective, and permanently open cohort of university graduates that began the recruitment of participants in 1999. Its design was based on other American large cohort studies. More detailed information about the Sun cohort has been previously described elsewhere [23]. Its primary aim was to assess the impact of lifestyle and diet on non-communicable diseases. Self-reported questionnaires at baseline and every 2 years are used to collect information mainly through mailed or electronically. The baseline questionnaire collects information related to socio-demographics variables, lifestyle, medical history, and anthropometric variables. 

**Figure 1:**
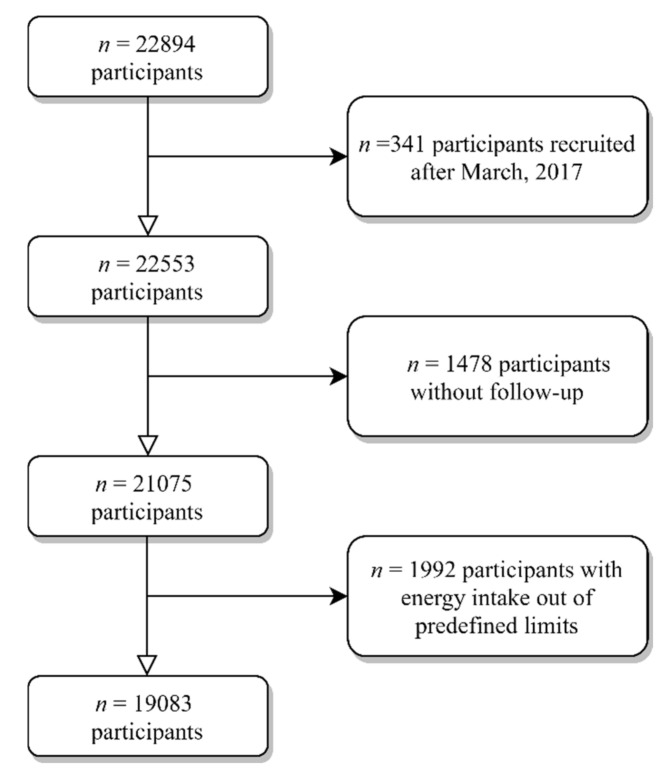
Flowchart of participants included in the study.

By December 2019, a total of 22,894 participants were recruited. For this study, individuals recruited after March 2017 (*n* = 341) were excluded to ensure a minimum follow-up of 2 years. We additionally excluded 1478 individuals without follow-up (retention rate 90.6%), and 1992 participants with energy intake outside of predefined limits (<500 or >3500 kcal/d for women, and <800 or >4000 kcal/d for men) [24] (Figure 1). Therefore, our analysis included a total of 19,083 subjects. 

At entry to the SUN project, participants receive detailed written information and give their permission to participate in the study before any follow-up on their medical history. The voluntary completion of questionnaire at baseline implies informed consent. According to the Declaration of Helsinki, potential candidates are informed of their right to drop out of the SUN project or withdraw their consent to continue in the study at any time without reprisal. The Institutional Review Board of the University of Navarra approved the SUN project, which is registered at clinicaltrials.gov (accessed on 2 February 2021) as NCT02669602.

### 2.2. Exposure Assessment

Participants provided their dietary intake information at the beginning of the study (baseline) and after 10 years of follow-up. A validated self-administrated semi-quantitative 136-item food-frequency questionnaire (FFQ) was used to assess participants ‘dietary intake [25,26,27]. The FFQ classifies food items into general food groups: dairy, eggs, meat and fish, vegetables, fruits, legumes and cereals, oils and fats, pastries, beverages, and miscellaneous. FFQ items included a standard portion size and 9 consumption frequency options, from “never/almost never” to “more than 6 times/day”. Daily consumption of every food item was estimated by multiplying its typical portion size with its frequency of consumption using an ad hoc computer program specifically developed for this aim. The calculation of dietary intakes was performed by a team of trained nutritionist using the daily intake of every food item and the corresponding nutrient composition based on Spanish Food Composition Tables [28,29]. Missing information on any dietary item was considered as having no consumption for such items. 

The MQI was calculated from dietary intake collected in the FFQ and was defined summing up three sub-indexes: the carbohydrate quality index (CQI), the fat quality index (FQI), and the healthy plate protein source quality index (HPPQI), as follows, MQI = CQI + FQI + HPPQI.

In brief, the CQI is based on 4 carbohydrate quality domains: glycemic index (GI), total dietary fiber intake (g/d), ratio of carbohydrates from solid/total (solid + liquid) carbohydrates, and ratio of carbohydrates from whole grains/carbohydrates from total grains (whole grains + refined grains or their products). Liquid carbohydrates were calculated summing up alcoholic and sugar-sweetened beverages and fruit juices consumption (4 items in the FFQ), while solid carbohydrate corresponded to the carbohydrate content of other foods with any carbohydrate content. On the other hand, refined grains were derived from the 13 items in the validated FFQ related to the consumption of white rice, refined bread, refined pasta, and different bakery products made with refined grains, and finally, whole grain consumption corresponded to the item “whole bread consumption” (serving size 60 g, 9 categories in the validated FFQ). The CQI was has been used in previous studies in the SUN Project, in the PREDIMED (Prevención con Dieta Mediterránea) and in the PREDIMED-Plus trials [30,31,32,33,34]. For each of its four components, we categorized participants into quintiles and received a value (ranging from 1 to 5) according to each quintile (only for GI the values were in reverse). Finally, we constructed the CQI summing for each participant the four values. The metabolomic signature of this dietary quality index has been specifically evaluated in the PREDIMED study [35].

To calculate the FQI as a continuous variable, we used the following ratio:

FQI = (Monounsaturated + Polyunsaturated)/(Saturated + Trans Fatty Acids) (MUFA + PUFA)/(SFA + TFA).

These four components were given the same weighting in the FQI. This dietary quality index has been previously used to investigate the association with nutritional adequacy in this cohort [31] and also in the PREDIMED study [33] and also with the risk of CVD [36] and with telomere length [37], among other outcomes.

Finally, the novel HPPQI has been created based on the nutritional protein quality recommendations found in the last international dietary guidelines [38,39,40,41]. This new dietary quality index was calculated with the following ratio:

HPPQI = (seafood + poultry + pulses + nuts)/(red and processed meats + cheese).

Thus, the foods located in the numerator and denominator reflect healthy and unhealthy source of proteins, respectively, according to the Harvard’s Healthy Eating Plate [41].

Lastly, to calculate each criterion of the MQI, the weighting of each criteria, CQI, FQI, and HPPQI was equal. We categorized participants into quintiles for each sub-index and we assigned values ranging from 1 point (first quintile) to 5 points (fifth quintile). Lastly, we calculated the MQI score adding the values of each sub-index, ranging from 3 (the worst quality) to 15 points (the best quality) (Table 1).

### 2.3. Outcome Assessment: All-Cause Mortality

The primary outcome was all-cause mortality. Information on mortality and its causes was identified through active and permanent contact with participants. Participants who did not respond to any of the 5 follow-up mailings were contacted by email or phone. We were able to identify approximately 75% of deaths through reports next-to skin, work associates, professional associations, or the authorities’ postal system. Additionally, we checked, at least once a year, both the National Statistical Institute and the Spanish National Death Index—a central index that collects information of any death registered in Spain, to confirm the status vital of all participants and to identify their cause of death if unknown. The last time when mortality and its causes were checked was December 2018. Death certificates and medical records of its causes were obtained.

### 2.4. Ascertainment of Covariates

When participants enter in the cohort, they provided additional information about socio-demographic characteristics (marital status and level of education, among others), anthropometric measures (weight, height, and weight change during the past 5 years), lifestyle and health-related habits (alcohol intake, smoking status, physical activity, leisure-time watching TV, lifestyle behavior, supplement use, and special diet), and family and personal medical history. Two subsamples of the SUN cohort were used to assess accuracy of physical activity [42] and of self-reported weight and height [43]. A 9-item Mediterranean Diet Score [44], frequently used in nutritional epidemiology [45] was used to assess the adherence to this dietary pattern. The prevalence and history of several non-communicable diseases was ascertained at baseline and updated until the exit of the cohort or until death was reported. Cardiovascular disease included any reported medical diagnosis of myocardial infarction, stroke, atrial fibrillation, coronary artery bypass grafting or other re-vascularization procedures, paroxysmal tachycardia, peripheral venous thrombosis, aortic aneurism, pulmonary embolism or heart failure. Self-reported cardiovascular events, cancer, and diabetes have always been confirmed by medical records [43,46,47]. These self-reports can be considered as reliable given than all participants of the SUN project are graduates, and more than 50% were health professionals themselves. Moreover, the validity of self-reported obesity, dyslipidemia, and hypertension diagnoses was evaluated in different subsamples of the SUN project.

### 2.5. Statistical Analyses

We described the baseline characteristics of participants adjusted for age and sex using the inverse probability weighting method, using proportions and means according to quartiles of the MQI. We use crude and multivariable Cox regressions models to assess the association of the MQI and each of its sub-index (CQI, FQI, and HPPQI) with all-cause mortality. We calculated hazard ratios (HRs) with their 95 % confidence interval (CIs) and we considered always the lowest quartile as the reference category.

All models included age (underlying time variable), and were stratified by recruitment period (5 categories) and deciles of age. Time at entry was considered as the date of completion of first questionnaire and exit time was date of death or date when participants completed the last follow-up questionnaire. We determined follow-up from the date when the questionnaire completed at baseline to the last follow-up questionnaire was returned, for whom death was not reported.

Participants completed a full-length FFQ after 10 years of follow-up allowed. We used repeated measurements using both cumulative average information and updated data of the MQI and its sub-indexes to reduce any effect of variation in dietary pattern and to obtain a more realistic measurement of long-term diet during follow-up.

After crude analyses, we fitted three multivariate Cox regression models with potential confounders that may affect the effect of CQI on mortality risk: model 1 was further adjusted for sex and total energy intake (kcal/d, continuous); model 2 was additionally adjusted for marital status (single, married, widowed, and others), educational level (years of higher education, continuous), smoking (never, current, and former smoker), cumulative smoking habit (packs/year, continuous), alcohol intake (never, <5 women or <10 men g/d, 5–25 women or 10–50 men g/d, and >25 women or >50 men g/d), leisure-time physical activity (metabolic equivalent-h/week, continuous), body mass index (BMI [kg/m^2^, linear and quadratic terms, continuous]), time spent sitting (hours/week, continuous), weight gain in the previous 5 years before entering the cohort (<3 kg and ≥3 kg), and following a special diet at baseline (yes/no); and model 3 was further adjusted for family history of CVD (yes/no), and the following prevalent diseases (yes/no): diabetes, CVD, cancer, depression, hypertension, hypercholesterolemia, and hypertriglyceridemia.

The individual contribution of each specific sub-index was also evaluated. Moreover, when we calculated HR for each component of MQI, we fitted an additional analysis adjusting for the intake of the rest of macronutrients in the fully adjusted model.

Potential confounders were selected a priori based on prior knowledge and previous findings of the SUN cohort on all-cause mortality [19,48]. We assessed linear trends across increasing categories by assigning medians to each quartile, and this variable was treated as continuous.

We assessed the interaction between age (continuous) and the MQI (continuous and categorized in quartiles) by testing an interaction product-term with the maximum likelihood ratio test.

The following sensitivity analyses were performed: (a) selection of only male or only female participants, (b) selection of participants aged <45 or ≥45 years, (c) censoring the analysis in ≥50 years, (d) selection of only health or non-health professionals, (d) excluding subjects with prevalent hypertension or hypercholesterolemia, (e) definition of a different predefined limits of energy intake (percentiles 5 and 95), (f) excluding subjects with prevalent CVD or cancer, (g) excluding subjects who were following a special diet at baseline, (h) excluding subjects with ≥30 missing items in FFQ, (i) redefining the MQI used the following ratio to calculate PQI_a_ = (fish + poultry and lean meats + eggs + pulses + nuts + reduced-fat dairy + whole grains)/(red and processed meats + full-fat dairy + refined cereals), (j) redefining the MQI used the following ratio to calculate PQI_b_ = (seafood + poultry + pulses + nuts + dairy products)/(red and processed meats) and finally (k) changing the weights of the 3 components of the MQI as follows, CQI (55%), FQI (30%) and HPPQI (15%) according to an acceptable macronutrient distribution [40].

We used in all analyses STATA version 16 (STATA Corp., College Station, TX, USA) with the SUN database updated in December 2019. All values presented are two-tailed; *p*-value < 0.05 was deemed as statistically significant.

## 3. Results

The present analysis included 19,083 Spanish adults (mean [SD] age 38.4 [12.4] years; 7654 men [40.5%]) that were followed up for a median time of 12.2 years (interquartile range 8.3 to 14.9). During this period, we identified 440 deaths from all causes, including 84 deaths due to CVD, 226 due to cancer, and 130 due to other causes. Among deceased participants, the mean age (SD) at death was 57.8 (15.5) years.

Table 2 shows age- and sex-adjusted baseline characteristics of participants according to quartiles of MQI. The median value of the MQI was 9 (range 3 to 15). Participants with higher MQI scores (range 12 to 15) were more likely to be single, never smokers, physically active, follow a special diet, and had higher baseline prevalence of chronic conditions. Individuals with better MQI scores had lower energy intake from fat. Conversely, participants with lower adherence to the MQI (range 3 to 7) were more likely to be married, and to snack be between meals and had lower adherence to a Mediterranean diet.

Table 3 presents the associations between all-cause mortality associated with quartiles of the MQI. No significant association was observed between mortality and the MQI when comparing the highest vs. the lowest quartile (reference category). In model 3, the adjusted HRs across successive quartiles compared with Q1 were: 1.03 (95% CI, 0.79–1.34) for Q2; 1.15 (95 % CI, 0.89–1.50) for Q3; and 0.79 (95% CI, 0.59–1.06) for Q4, with a non-significant linear trend (P_trend_ = 0.199). We did not find any interaction between age and MQI (continuous, *p*-interaction = 0.640 and categorized in quartiles, *p*-interaction = 0.451).

The relationship between quartiles of each individual dimension of the MQI and mortality was additionally assessed (Table 4). The multivariate-adjusted HR between extreme quartiles of the CQI was 0.64 (95% CI, 0.45–0.90), with a significant inverse dose-response relation (P_trend_ = 0.021). However, neither FQI nor HPPQI were associated with lower all-cause mortality risk. For the FQI, the multivariable-adjusted HRs were 0.85 (95% CI, 0.64–1.13), 0.86 (95% CI, 0.65–1.14) and 0.75 (95% CI, 0.56–1.00) in successive quartiles in comparison with the lowest quartile (P_trend_ = 0.070). On the other hand, for the HPPQI the HRs in the most adjusted model were: 0.80 (95% CI, 0.60–1.07), 0.74 (95% CI, 0.55–0.99) and 0.93 (95% CI, 0.69–1.25) for the Q2, Q3, and Q4 respectively (P_trend_ = 0.844).

We conducted Cox regression models to calculate the risk of mortality, comparing quartiles of MQI (Table 5) and CQI (Table 6) for update and cumulative average dietary information after 10 years of follow-up. After using repeated nutritional measurements in our analyses, we found that the MQI was not associated with lower mortality when comparing extreme quartiles. Thus, the MQI was not associated with significantly different rates of all-cause mortality with HRs of 0.90 (95% CI, 0.66–1.23) when using an Updated Diet and 0.84 (95% CI, 0.63–1.12) when using Cumulative Diet Average with the information repeated after 10-year follow-up. However, the individual analysis of CQI revealed an inverse association with all-cause of mortality when we compared extreme quartiles using both approaches: multivariable-adjusted HRs 0.68 (95% CI, 0.49–0.94; P_trend_ = 0.031) for updated, and 0.69 (95% CI, 0.50–0.93; P_trend_ = 0.016) for cumulative average information, respectively.

**Figure 2:**
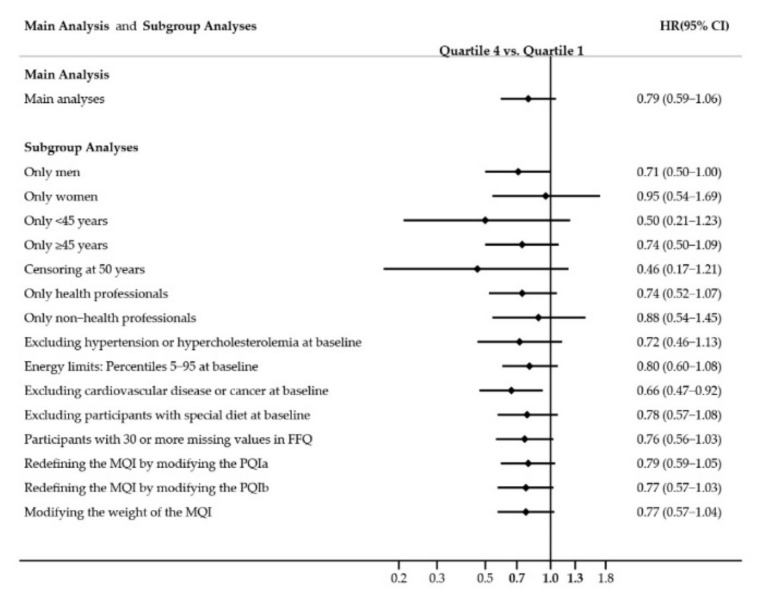
Hazard ratios (HR) and 95% confidence intervals (CI) between extreme quartiles for the association between Macronutrient Quality Index (MQI) and mortality in the SUN cohort. Abbreviations: FFQ, food frequency questionnaire, MQI, Macronutrient Quality Index; HPPQI, Healthy Plate Protein Source Quality Index. PQIa was defined as the following ratio: (fish + poultry and lean meats + eggs + pulses + nuts + reduced-fat dairy + whole grains)/(red and processed meats + full-fat dairy + refined cereals); PQIb was defined as the following ratio: (seafood + poultry + pulses + nuts + dairy products)/(red and processed meats).

We conducted multiple sensitivity analyses to assess the robustness of our findings. Overall, these analyses were consistent with our main analyses and estimates remained similar (Figure 2).

## 4. Discussion

In this Mediterranean population, we found no statistically significant association between a new multidimensional score of macronutrient quality, MQI, and all-cause mortality. However, higher quality of carbohydrate showed an association with lower mortality.

The role of carbohydrate intake on health continues to be a matter of debate. Concerning quantity, a previous investigation found a U-shaped association between carbohydrate intake and mortality [8]. Nevertheless, other authors have suggested that carbohydrate quality and food sources seem to play a more important role in population health than quantity of carbohydrate intake [9,20]. In the majority of the studies, carbohydrate quality has been operationally measured using unidimensional indicators, mainly dietary GI or glycemic load, dietary fiber, or whole grains intake. In fact, both higher intakes of whole grains and dietary fiber are associated with reduced incidence of mortality, whereas less useful markers of carbohydrate quality on mortality are GI/load [21,49,50]. Furthermore, higher consumption of starchy carbohydrates and sugar are associated with a higher mortality risk [51]. Possible biological explanation for this association is that higher fiber intake could reduce concentrations of serum inflammatory biomarkers, and inflammation could be associated with mortality risk [22,52]. Previous investigation in this cohort, conclude that CQI seems to reflect the combined effect and potential synergies of different dimensions of carbohydrate quality [19].

Regarding fat quality, we used the ratio MUFA + PUFA /SFA + TFA. This criterion was based on current dietary guidelines and previous evidence on the association of diets high in SFA and TFA and with higher mortality, whereas in the case of diets high in PUFA and MUFA, there was an inverse association [14]. In particular, the anti-inflammatory effect of omega-3 fatty acids could explain, at least partly, their beneficial effect on mortality. Furthermore, the pathophysiology of neurodegenerative or CVD, cancers, among other age-related morbidities, has been associated with inflammation [53]. However, in our study, FQI was not independently associated with all-cause mortality. A previous large research work including 18 countries found that higher intake of MUFA, PUFA, and SFA was associated with lower mortality risk [12]. In this sense, emerging evidence is questioning current dietary fat recommendations, especially in case of SFA and food sources of specific fatty acids, arguing that SFA can have different biologic effects, which could be further modified by the carbohydrate content of diet food matrix [13,51]. Some evidence suggests that SFA (and especially dairy-derived SFA) [54], may not predict overall mortality [13,55] and that MUFA may also be not relevant, since, just as an example, a PREDIMED cohort derived paper, comparing extra-virgin and normal olive oil association with CVD, yields opposite results, suggesting that MUFA may have no direct effect on this outcome, be just be a marker of the intake of some phytochemical (polyphenols) [56,57]. However, isocaloric substitution models [58,59] and the 2015 meta-analysis by the Cochrane Collaboration [60] support small, but potentially important, reductions in CVD and total mortality by reducing SFA intake, and replacing it with PUFA or MUFA. Replacement with carbohydrate appears less useful.

In relation to protein quality, higher HPPQI was not associated with lower mortality risk in this study. Previous investigations on the influence of protein in all-cause mortality have centered on animal or plant proteins effect, or replacement of animal sources by plant sources [15,17]. In general, protein-rich diets, particularly including red and processed meats, and also animal sources increase health risks and mortality [17,61]. On the other hand, among elderly people, higher protein intake is crucial to increase muscle mass and strength, which are both independent predictors of mortality [53,62]. Regarding protein quality intake, food content of essential amino acids and their digestibility, have been widely used as traditional indicators, but in this context, food sources of traditional high-quality protein, do not imply higher diet quality or health outcomes. For instance, certain animal sources of protein have been related to increased morbidity, whereas consumption of plant proteins and plant-based dietary patterns are associated with more health benefits [63]. As mentioned before, in our investigation, protein quality was established, taking into account food protein sources, classified into healthy or unhealthy, independently of its animal or plant origin. Thus, the healthy protein group included both animal (seafood and poultry). In relation to meat and dairy, which are sources of SFA, TFA, and protein, but also micronutrients, Astrup et al. [13], found these food groups are not associated with increased risk of CVD, and suggested that limiting their intake is not supported by recent evidence. In this sense, the complexity and diversity of the relation between isolated or global macronutrient intake, sources, and health outcomes, should be highlighted. Thus, health effects of foods may not be predicted by their content in any nutrient group without considering the overall macronutrient distribution and food matrix [51].

Several limitations should be acknowledged. First, dietary variables were self-reported through a FFQ, which could have a certain degree of measurement bias. However, this method is considered the most appropriate approach to assess food and nutrient intake in large cohorts and moreover, the FFQ used in this adult cohort, has been repeatedly validated [25,26]. Besides, participants with energy intakes outside predefined limits were excluded [24]. Second, habitual diet was assessed at baseline, and changes in diet during follow-up are possible. For this reason, we repeated the main analysis after 10-years of follow-up using two different approaches to better capture the exposure. Third, the dietary quality indexes were not formally validated as such, but we have calculated them through a FFQ repeatedly validated in Spain [25,26,27]. Fourth, our sample was not representative of the general population. Thus, the generalization of our findings should be based on plausible biological mechanisms rather than on statistical representativeness. However, this homogeneity among participants reduces the likelihood of misclassification bias, reduces potential confounding and increase internal validity. Fifth, this is cohort of university graduates, which may influence diet and lifestyles. However, both high level of education and homogeneity of the participants add validity to the self-reported data collection and prevent confounding by education and other socioeconomic factors. Sixth, residual confounding cannot be totally eliminated, even though analyses were adjusted for major potential confounders. Finally, this is a young cohort with low rate of deaths that may have affected the main analyses. Thus, our estimates for all-cause mortality could be underpowered. Seventh, we acknowledge that the MQI and its components have not been formally validated, and their building might raise some concerns about arbitrary decisions. However, we feel that such concerns are unfounded because we defined all indices a priori, meaning that we used previously peer-reviewed published literature to compute them. Therefore, the arbitrariness of indices should not be claimed. In fact, we created our indices based on the best scientific evidence available at the moment of conducting our analyses. In mainstream nutritional science, most a priori-defined dietary indexes assign equal weights to each item. For example, three a priori dietary quality indexes have used our same approach to equally weight each component: the Provegetarian Food Pattern [64], the Mediterranean Diet Score [44], and the Dietary approaches to Stop Hypertension [65].

To date, overall quality of macronutrient intake has not been prospectively assessed at the same time in a large adult cohort. Furthermore, this study includes the use of the HPPQI, a novel index to measure protein quality, and the MQI have been calculated using dietary quality indexes, the FQI and the CQI, repeatedly used in previous research. Other strengths are the relatively large sample size, its prospective design with along follow-up period, the high retention proportion, and the use of multivariate analyses, adjusted for a wide number of potential confounders. Besides, additional assets of this investigation are the use of validated questionnaires and methods, the utilization of repeated dietary measurements and the inclusion of multiple sensitivity analyses, which adds robustness to findings. It has to be also mentioned the verification of deaths by medical records or consultation of the National Death Index. Finally, to our knowledge, there are not previous studies that have examined the association of a global MQI with all-cause mortality.

## 5. Conclusions

In conclusion, in this Mediterranean cohort, we did not find a significant inverse relationship between a new multidimensional MQI and lower risk of all-cause mortality, but the association was significant for the CQI. These results emphasize the difficulty in establishing associations between global quality of macronutrients and mortality risk among middle-aged adults.

## Figures and Tables

**Table 1:** Components of the Macronutrient Quality Index.

Components of Macronutrient Quality Index	Index Range (Points)	Criteria for Minimum Index	Criteria for Maximum Index
Carbohydrate quality index (CQI)	1–5	Minimum CQI (first quintile)	Maximum CQI (fifth quintile)
Fat quality index (FQI)	1–5	Minimum FQI (first quintile)	Maximum FQI (fifth quintile)
Healthy Plate Protein quality index (HPPQI)	1–5	Minimum HPPQI (first quintile)	Maximum HPPQI (fifth quintile)
Total index (range)	3–15		

**Table 2:** Age and sex-adjusted baseline characteristics of participants according to quartiles of the Macronutrient Quality Index (MQI): the SUN (Seguimiento Universidad de Navarra) cohort ^1,2^.

Characteristics	Q1	Q2	Q3	Q4
*n* (frequency)	6646	4509	3906	4022
MQI range	<8	8–9	10–11	>11
Marital status				
Single	42.8	43.4	43.4	45.9
Married	52.1	50.8	50.9	47.5
Widowed	1.0	1.2	1.0	0.8
Others	4.2	4.7	4.7	5.7
Years at university	5.1 (1.5)	5.1 (1.6)	5.0 (1.5)	5.0 (1.5)
Smoking (%)				
Never smoker	47.0	46.0	49.6	49.4
Current smoker	28.6	28.7	29.7	32.0
Former smoker	24.4	25.3	20.7	18.6
Cumulative smoking habit (packs-years)	6.1 (10.1)	6.1 (10.1)	5.7 (9.6)	5.5 (9.1)
Alcohol intake (g/d)				
Never	18.5	17.3	17.3	18.0
<5 women/<10 men	48.4	48.4	48.3	49.4
5–25 women/10–50 men	31.3	32.2	32.3	31.2
>25 women/>50 men	1.9	2.1	2.1	1.4
Physical activity (METs-h/week)	19.1 (20.7)	20.7 (21.8)	23.0 (24.5)	26.3 (25.9)
Body mass index (kg/m^2^)	23.5 (3.6)	23.6 (3.5)	23.7 (3.6)	23.5 (3.5)
Time spent sitting (h/d)	5.3 (2.1)	5.3 (2.0)	5.2 (2.0)	5.2 (2.0)
Snacking between meals (% Yes)	35.4	34.3	31.5	29.5
Special diet (% Yes)	5.2	7.1	9.3	14.6
Medically-diagnosed conditions at baseline				
Prevalent diabetes (%)	1.6	2.0	2.5	2.2
Prevalent hypertension (%)	10.3	10.8	11.5	12.7
Prevalent dyslipemia (%)	6.1	6.9	7.7	7.8
Prevalent cardiovascular disease (%) ^3^	4.5	4.2	4.9	5.0
Prevalent cancer (%)	2.6	2.4	2.4	2.8
Dietary variables				
Trichopoulou’s MedDiet score	2.6 (1.3)	3.6 (1.3)	4.4 (1.3)	5.3 (1.3)
Total energy intake (kcal/d)	2304 (613)	2355 (618)	2369 (633)	2364 (620)
Carbohydrate intake, % E	41.7 (7.3)	43.0 (7.1)	43.9 (7.2)	45.6 (7.8)
Fat intake, % E	37.8 (6.1)	36.7 (6.3)	35.9 (6.6)	34.3 (7.0)
Protein intake, % E	18.4 (3.3)	18.2 (3.1)	18.1 (3.5)	18.2 (3.5)

Abbreviations: MQI, Macronutrient Quality Index; Q, quartile; %E, percentage of energy intake. ^1^ Adjusted through inverse probability weighting. ^2^ Values are means ± SDs or numbers of participants (percentages) unless otherwise indicated. ^3^ Prevalent CVD was considered as any of the following medical diagnosis occurring prior to entering in the study: myocardial infarction, stroke, atrial fibrillation, coronary artery bypass grafting or other re-vascularization procedures, paroxysmal tachycardia, peripheral venous thrombosis, aortic aneurism, pulmonary embolism or heart failure.

**Table 3:** Hazard ratios (HR) and 95% confidence intervals (CI) for the association between quartiles of the Macronutrient Quality Index (MQI) and all-cause mortality in the SUN cohort (*n* = 19,083).

	Q1	Q2	Q3	Q4	*p* for Trend
*n* (frequency)	6646	4509	3906	4022	
MQI range	<8	8–9	10–11	>11	
Deaths	138	102	113	87	
Person-years	79,526	52,396	44,304	43,661	
Mortality rate/1000 person years	1.74	1.95	2.55	1.99	
Crude model	1.00 (Ref.)	1.10 (0.85–1.42)	1.43 (1.12–1.84)	1.10 (0.84–1.44)	0.214
Model 1	1.00 (Ref.)	0.98 (0.75–1.27)	1.10 (0.85–1.42)	0.71 (0.54–0.94)	0.035
Model 2	1.00 (Ref.)	1.07 (0.83–1.39)	1.20 (0.93–1.56)	0.84 (0.63–1.12)	0.408
Model 3	1.00 (Ref.)	1.03 (0.79–1.34)	1.15 (0.89–1.50)	0.79 (0.59–1.06)	0.199

Abbreviations: MQI, Macronutrient Quality Index; Q, quartile; Ref., referent value. Model 1: adjusted for sex (dichotomous), age (underlying variable), total energy intake (continuous), and stratified by recruitment period (5 categories), and deciles of age. Model 2: additionally adjusted for marital status (four categories), educational level (years of higher education, continuous), smoking (three categories), cumulative smoking habit (packs/year, continuous), alcohol intake (four categories), leisure-time physical activity (metabolic equivalent-h/week, continuous), BMI (kg/m^2^, linear, continuous), time spent sitting (hours/week, continuous), weight gain in the previous 5 years before entering the cohort (dichotomous) and following a special diet at baseline (dichotomous). Model 3: additionally adjusted for family history of CVD (dichotomous), and prevalent diagnosis of diabetes (dichotomous), hypertension (dichotomous), hypercholesterolemia (dichotomous), hypertriglyceridemia (dichotomous), CVD (dichotomous), depression (dichotomous), and cancer (dichotomous).

**Table 4:** Hazard ratios (HR) and 95% confidence intervals (CI) for the association between quartiles of the Carbohydrate Quality Index (CQI), Fat Quality Index (FQI), Protein Quality Index (PQI), and all-cause mortality in the SUN cohort (*n* = 19,083).

CQI	Q1	Q2	Q3	Q4	*p* for Trend
*n* (frequency)	6038	4281	5366	3398	
CQI range	<10	10–11	12–14	>14	
Deaths	139	109	129	63	
Person-years	71,607	50,014	60,962	37,303	
Mortality rate/1000 person years	1.94	2.18	2.12	1.69	
Crude model	1.00 (Ref.)	1.12 (0.87–1.44)	1.06 (0.83–1.35)	0.84 (0.62–1.13)	0.337
Model 1	1.00 (Ref.)	0.87 (0.67–1.12)	0.86 (0.67–1.10)	0.57 (0.42–0.78)	0.001
Model 2	1.00 (Ref.)	0.98 (0.75–1.27)	0.97 (0.75–1.25)	0.66 (0.48–0.91)	0.024
Model 3	1.00 (Ref.)	0.99 (0.76–1.28)	0.99 (0.77–1.28)	0.65 (0.47–0.90)	0.023
Model 4	1.00 (Ref.)	0.97 (0.74–1.27)	0.96 (0.73–1.27)	0.64 (0.45–0.90)	0.021
**FQI**	**Q1**	**Q2**	**Q3**	**Q4**	***p* for Trend**
*n* (frequency)	4771	4771	4771	4770	
FQI range	<1.40	1.40–1.61	1.62–1.90	>1.90	
Deaths	116	93	108	123	
Person-years	55,424	56,039	54,355	54,069	
Mortality rate/1000 person years	2.10	1.66	1.99	2.27	
Crude model	1.00 (Ref.)	0.78 (0.59–1.03)	0.94 (0.72–1.22)	1.07 (0.83–1.37)	0.276
Model 1	1.00 (Ref.)	0.90 (0.69–1.19)	0.90 (0.69–1.18)	0.91 (0.70–1.18)	0.547
Model 2	1.00 (Ref.)	0.93 (0.70–1.24)	0.99 (0.75–1.30)	0.94 (0.72–1.22)	0.723
Model 3	1.00 (Ref.)	0.89 (0.67–1.18)	0.92 (0.70–1.21)	0.86 (0.66–1.12)	0.337
Model 5	1.00 (Ref.)	0.85 (0.64–1.13)	0.86 (0.65–1.14)	0.75 (0.56–1.00)	0.070
**HPPQI**	**Q1**	**Q2**	**Q3**	**Q4**	***p* for Trend**
*n* (frequency)	4771	4771	4771	4770	
PQI range	<0.65	0.65–0.93	0.93–1.38	>1.38	
Deaths	115	87	103	135	
Person-years	58,047	55,988	53,819	52,032	
Mortality rate/1000 person years	1.98	1.55	1.91	2.59	
Crude model	1.00 (Ref.)	0.77 (0.59–1.02)	0.93 (0.71–1.21)	1.26 (0.98–1.61)	0.007
Model 1	1.00 (Ref.)	0.71 (0.54–0.94)	0.72 (0.55–0.94)	0.82 (0.63–1.05)	0.470
Model 2	1.00 (Ref.)	0.81 (0.61–1.08)	0.79 (0.60–1.04)	0.97 (0.74–1.25)	0.781
Model 3	1.00 (Ref.)	0.79 (0.60–1.05)	0.73 (0.55–0.97)	0.92 (0.70–1.19)	0.945
Model 6	1.00 (Ref.)	0.80 (0.60–1.07)	0.74 (0.55–0.99)	0.93 (0.69–1.25)	0.844

Abbreviations: Ref., referent value; Q, quartile. Model 1: adjusted for sex (dichotomous), age (underlying variable), total energy intake (continuous) and stratified by recruitment period (5 categories) and deciles of age. Model 2: additionally adjusted for marital status (four categories), educational level (years of higher education, continuous), smoking (three categories), cumulative smoking habit (package/year, continuous), alcohol intake (four categories), leisure-time physical activity (metabolic equivalent-h/week, continuous), BMI (kg/m^2^, linear, continuous), time spent sitting (hours/week, continuous), weight gain in the previous 5 years before entering the cohort (dichotomous) and following a special diet at baseline (dichotomous). Model 3: additionally adjusted for family history of CVD (dichotomous), and prevalent diagnosis of diabetes (dichotomous), hypertension (dichotomous), hypercholesterolemia (dichotomous), hypertriglyceridemia (dichotomous), CVD (dichotomous), depression (dichotomous), and cancer (dichotomous). Model 4: additionally adjusted for snacking (dichotomous), animal protein (g/d, continuous), plant protein (g/d, continuous), MUFA (%E, continuous), PUFA (%E, continuous), SFA (%E, continuous), and TFA (%E, continuous). Model 5: additionally adjusted for snacking (dichotomous), animal protein (g/d, continuous), plant protein (g/d, continuous), and carbohydrate intake g/d, continuous). Model 6: additionally adjusted for snacking (dichotomous), MUFA (%E, continuous), PUFA (%E, continuous), SFA (%E, continuous), TFA (%E, continuous), and carbohydrate intake (g/d, continuous).

**Table 5:** Updated ^1^ and cumulative average ^2^ dietary measurements after 10 years of follow-up. Hazard ratios (HR) and 95% confidence intervals (CI) for the association between quartiles of the Macronutrient Quality Index (MQI) and mortality in the SUN cohort.

Updated Diet ^1^	Q1	Q2	Q3	Q4	*p* for Trend
MQI range	<7	7–9	10–11	>11	
Deaths	93	149	107	91	
Person-years	56,297	77,428	42,972	43,190	
Mortality rate/1000 person years	1.65	1.92	2.49	2.11	
Crude model	1.00 (Ref.)	1.18 (0.91–1.53)	1.53 (1.16–2.02)	1.25 (0.94–1.67)	0.044
Model 1	1.00 (Ref.)	1.04 (0.80–1.36)	1.14 (0.86–1.52)	0.81 (0.60–1.10)	0.211
Model 2	1.00 (Ref.)	1.11 (0.85–1.44)	1.25 (0.93–1.67)	0.97 (0.71–1.32)	0.953
Model 3	1.00 (Ref.)	1.06 (0.81–1.38)	1.19 (0.89–1.60)	0.90 (0.66–1.23)	0.610
**Cumulative Diet Average ^2^**	**Q1**	**Q2**	**Q3**	**Q4**	***p* for Trend**
MQI range	>7.5	7.5–9	9.5–11	>11	
Deaths	137	104	110	89	
Person-years	79,578	53,326	44,228	42,754	
Mortality rate/1000 person years	1.72	1.95	2.49	2.08	
Crude model	1.00 (Ref.)	1.10 (0.85–1.42)	1.41 (1.10–1.81)	1.17 (0.89–1.52)	0.095
Model 1	1.00 (Ref.)	0.97 (0.75–1.25)	1.09 (0.84–1.41)	0.75 (0.57–0.99)	0.079
Model 2	1.00 (Ref.)	1.06 (0.82–1.38)	1.19 (0.92–1.55)	0.90 (0.67–1.20)	0.630
Model 3	1.00 (Ref.)	1.02 (0.78–1.33)	1.16 (0.89–1.51)	0.84 (0.63–1.12)	0.367

Abbreviations: Ref., referent value; Q, quartile. ^1^ Repeated measures: update information of MQI after 10 years of follow-up. ^2^ Repeated measures: cumulative average information of MQI (at baseline and after 10 years of follow-up). Model 1: adjusted for sex (dichotomous), age (underlying variable), total energy intake (continuous) and sex (dichotomous) stratified by recruitment period (5 categories) and deciles of age and recruitment period (5 categories). Model 2: additionally adjusted for marital status (four categories), educational level (years of higher education, continuous), smoking (three categories), cumulative smoking habit (package/year, continuous), alcohol intake (four categories), smoking (three categories), smoking habit (package/year, continuous), educational level (years of higher education, continuous), leisure-time physical activity (metabolic equivalent-h/week, continuous), BMI (kg/m^2^, linear, continuous), marital status (four categories), time spent sitting (hours/week, continuous), weight gain in the previous 5 years before entering the cohort (dichotomous) and following a special diet at baseline (dichotomous). Model 3: additionally adjusted for family history of CVD (dichotomous), and prevalent diagnosis of diabetes (dichotomous), hypertension (dichotomous), hypercholesterolemia (dichotomous), hypertriglyceridemia (dichotomous), CVD (dichotomous), depression (dichotomous), and cancer (dichotomous).

**Table 6:** Updated ^1^ and cumulative average ^2^ dietary measurements after 10 years of follow-up. Hazard ratios (HR) and 95% confidence intervals (CI) for the association between quartiles of the Carbohydrate Quality Index (CQI) and mortality in the SUN cohort.

Updated Diet ^1^	Q1	Q2	Q3	Q4	*p* for Trend
CQI range	<10	10–11	12–14	>14	
Deaths	136	108	129	67	
Person-years	69,854	48,971	62,043	39,018	
Mortality rate/1000 person years	1.95	2.21	2.08	1.71	
Crude model	1.00 (Ref.)	1.13 (0.87–1.45)	1.02 (0.80–1.29)	0.82 (0.61–1.10)	0.194
Model 1	1.00 (Ref.)	0.92 (0.69–1.16)	0.87 (0.68–1.12)	0.60 (0.44–0.81)	0.002
Model 2	1.00 (Ref.)	1.00 (0.77–1.30)	0.97 (0.75–1.25)	0.69 (0.51–0.94)	0.031
Model 3	1.00 (Ref.)	1.01 (0.78–1.31)	1.00 (0.77–1.29)	0.68 (0.50–0.93)	0.032
Model 4	1.00 (Ref.)	1.00 (0.76–1.31)	0.97 (0.74–1.28)	0.68 (0.49–0.94)	0.031
**Cumulative Diet Average ^2^**	**Q1**	**Q2**	**Q3**	**Q4**	***p* for Trend**
CQI range	<9.5	9.5–11	11.5–13.5	>13.5	
Deaths	133	111	103	93	
Person-years	68,635	51,152	46,443	43,657	
Mortality rate/1000 person years	1.94	2.17	2.21	1.73	
Crude model	1.00 (Ref.)	0.88 (0.68–1.14)	0.93 (0.72–1.21)	0.59 (0.46–0.78)	<0.001
Model 1	1.00 (Ref.)	0.89 (0.69–1.16)	0.94 (0.72–1.23)	0.61 (0.46–0.80)	0.001
Model 2	1.00 (Ref.)	1.01 (0.78–1.32)	1.04 (0.79–1.37)	0.71 (0.53–0.94)	0.017
Model 3	1.00 (Ref.)	1.02 (0.79–1.33)	1.07 (0.82–1.41)	0.70 (0.53–0.94)	0.017
Model 4	1.00 (Ref.)	1.01 (0.77–1.32)	1.05 (0.79–1.39)	0.69 (0.50–0.93)	0.016

Abbreviations: Ref., referent value; Q, quartile. ^1^ Repeated measures: update information of MQI after 10 years of follow-up. ^2^ Repeated measures: cumulative average information of CQI (at baseline and after 10 years of follow-up). Model 1: adjusted for sex (dichotomous), age (underlying variable), total energy intake (continuous) and stratified by recruitment period (5 categories) and deciles of age. Model 2: additionally adjusted for marital status (four categories), educational level (years of higher education, continuous), smoking (three categories), cumulative smoking habit (package/year, continuous), alcohol intake (four categories), leisure-time physical activity (metabolic equivalent-h/week, continuous), BMI (kg/m^2^, linear, continuous), time spent sitting (hours/week, continuous), weight gain in the previous 5 years before entering the cohort (dichotomous) and following a special diet at baseline (dichotomous). Model 3: additionally adjusted for family history of CVD (dichotomous), and prevalent diagnosis of diabetes (dichotomous), hypertension (dichotomous), hypercholesterolemia (dichotomous), hypertriglyceridemia (dichotomous), CVD (dichotomous), depression (dichotomous), and cancer (dichotomous). Model 4: additionally adjusted for snacking (dichotomous), animal protein (g/d, continuous), plant protein (g/d, continuous), MUFA (%E, continuous), PUFA (%E, continuous), SFA (%E, continuous), and TFA (%E, continuous).

## Data Availability

The data that support the findings of this study are available from the SUN Project at sun@unav.es, upon reasonable request.
